# Soft Robotics: New Perspectives for Robot Bodyware and Control

**DOI:** 10.3389/fbioe.2014.00003

**Published:** 2014-01-30

**Authors:** Cecilia Laschi, Matteo Cianchetti

**Affiliations:** ^1^The BioRobotics Institute, Scuola Superiore Sant’Anna, Pisa, Italy

**Keywords:** soft robotics, morphological computation, biomimetic robotics, biorobotics, smart materials

## Abstract

The remarkable advances of robotics in the last 50 years, which represent an incredible wealth of knowledge, are based on the fundamental assumption that robots are chains of rigid links. The use of soft materials in robotics, driven not only by new scientific paradigms (biomimetics, morphological computation, and others), but also by many applications (biomedical, service, rescue robots, and many more), is going to overcome these basic assumptions and makes the well-known theories and techniques poorly applicable, opening new perspectives for robot design and control. The current examples of soft robots represent a variety of solutions for actuation and control. Though very first steps, they have the potential for a radical technological change. Soft robotics is not just a new direction of technological development, but a novel approach to robotics, unhinging its fundamentals, with the potential to produce a new generation of robots, in the support of humans in our natural environments.

## The Soft Robotics Challenge

Robotics has grown exponentially in the last 50 years and today robotics technologies are very solid and robust, in the accurate, fast, and reliable control of robot motion. Almost all the theories and techniques for robot control, fabrication, and sensing, which represent an incredible wealth of knowledge, are based on a fundamental assumption and conventional definition of robots: a kinematic chain of rigid links.

Recent advances in soft and smart materials, compliant mechanisms, and non-linear modeling, on the other hand, have led to a more and more popular use of soft materials in robotics worldwide. This is driven not only by new scientific paradigms (biomimetics, morphological computation, and others) but also by many application requirements (in the fields of biomedical, service, rescue robots, and many more), because of the expected capability of soft robots to interact more easily and effectively with real-world environments (Mazzolai et al., 2012; Pfeifer et al., 2012).

In biomimetics, the use of soft materials is suggested by the uncountable examples of animal and vegetal systems. Rigid structures, like skeletons or exoskeletons, are always accompanied by soft tissues. These include mechanisms for varying the material characteristics such as stiffness, elasticity, and surface properties, etc. (Kim et al., 2013) for generating motion through muscles and for facilitating sensing in skins through embedded mechanoreceptors.

Compliance, or softness, is also needed for implementing the principles of embodied intelligence, or morphological computation, a modern view of intelligence, attributing a stronger role to the physical body and its interaction with the environment. It is current thinking in robotics that fast, efficient, and robust behavior can be achieved by adequately exploiting material properties and in particular softness (Pfeifer et al., 2012), and that soft materials may enable us to automate tasks that are beyond capacities of current robotics technology. The importance of soft body parts appears clear in natural organisms, to increase adaptability and robustness. For example, skin is soft and deformable, while at the same time it is robust and waterproof, and it is evident that it has a significant role in grasping and manipulation.

Indeed, the use of soft deformable and variable stiffness technologies in robotics represents an emerging approach to build new classes of robotic systems that are expected to interact more safely with the natural, unstructured environment and with humans, and that better deal with uncertain and dynamic tasks [i.e., grasping and manipulation of unknown objects (Brown et al., 2010), locomotion in rough terrains (Lin et al., 2011), physical contacts with human bodies, etc.].

The field of soft robotics is growing worldwide, but it is worth noticing that when speaking about “soft robotics,” two major approaches should be distinguished in providing soft interaction: (1) control of the actuator stiffness of robot with rigid links (Albu-Schäffer et al., 2008) and (2) softness intrinsically due to the passive characteristics of the robot bodyware (Trivedi et al., 2008).

In the first approach, robots are built with traditional rigid links, but the control system varies the resistance that the robot has to show at the interaction with the environment (objects or people), either through compliance or impedance control schemes (Siciliano and Villani, 1999). In rehabilitation, the robots used for physical therapy are controlled with interaction control schemes, which regulate their stiffness in accordance with the forces applied by the patients (Krebs et al., 2000). Still following the first approach, actuators are designed in order to have variable impedance. So-called variable impedance actuators (VIA) can show a behavior where the output stiffness can vary independently from the output position (Vanderborght et al., 2009; Visser et al., 2011).

In the second approach, the robots are made of soft materials and they undergo high deformations during interaction. In this different method, soft actuators and materials which can vary their stiffness are used, and their control is partially embedded in the body morphology. This approach exploits the material properties of the robot and its capacity to interact with the environment (Brooks, 1991; Pfeifer and Bongard, 2007). Soft-bodied robots are able to perform relatively large deformations under typical loading conditions and can exploit the passive deformations of the body to adjust to the environment (Brown et al., 2010). Thus, the tasks that in the classical approach are performed by the control system can be made partially redundant by the mechanical properties of the physical body itself (Pfeifer and Bongard, 2007). The main advantage is that the complex, precise control architecture can be simplified using highly compliant materials with variable stiffness, where the control is in part embedded in the morphology of the body, and the robot interactions with objects or the environment derive from the adaptability of the agent itself. This represents the base of the Morphological Computation theory.

Soft robotics is intended here in this second meaning, i.e., the use of soft materials and the implementation of mechanisms for varying the body shape and stiffness. This is a radically transformative approach, because it abandons the basic assumptions of robotics. Overcoming these assumptions means that well-known robotics theories and techniques are poorly applicable and that new solutions are needed.

We identify few challenges in the design and control of soft robots, some suitable technological solutions, and possible approaches, as described in the following.

## Smart Actuators and Manufacturing Technologies for Soft Robots

Within the framework of soft robotics, it is easy to understand how one of the most important bottleneck in developing effective robots is the lack of reliable and robust soft actuators. Nevertheless, new and promising technologies are emerging, attracting the attention of an increasing number of research groups. New smart materials with the same common denominator of softness or flexibility represent the next frontier in the development of soft actuators.

Shape Memory Alloys (SMAs) are metal alloys capable of undergoing a certain strain, and subsequently recover their original shape when heated. SMAs allow to drastically reduce the size, weight, and complexity of robotic systems. In fact, their large force–weight ratio, large life cycles, negligible volume, sensing capability, and noise-free operation enable the employment of this technology in soft robotics (Cianchetti, 2013). On the other side, they usually require relatively high currents and the transduction process is not highly efficient. Moreover, the high non-linearity and hysteresis associated to the material activation make SMAs very difficult to be precisely controlled.

Part of these drawbacks are avoided by using Shape Memory Polymers (SMPs), which exploit the same principle as SMAs but using different kind of stimuli (other than electricity) to trigger the activation. Chemical or thermal stimuli, light, and magnetic fields are the most used, demonstrating a higher transduction efficiency, but to the cost of an increased response time. SMPs belong to a class of smart polymers, which have drawn considerable research interest in the last few years because of their applications in micro-electromechanical systems and actuators in biomedical devices. In several fields of applications, SMPs’ materials have been proved to be suitable substitutes to metallic ones because of their flexibility, biocompatibility, and wide scope of modifications. A comprehensive review can be found in Ratna and Karger-Kocsis (2008).

Electro Active Polymers (EAPs) are a new emerging and promising class of technologies, which already demonstrated the possibility to fill the gap between natural and artificial muscles. Most of them are based on polymeric matrices activated with different mechanisms, but they are all endowed with the capability of varying their size and shape when an electric stimulus is supplied (Mirfakhrai et al., 2007). They have power densities exceeding those of biological muscle, are readily scalable and free-form fabricable, and are ideally suited to biomimetic and biomedical soft robotic applications. On the other side, depending on the specific EAP technology, slow response or high voltages request can limit their usability. Moreover, reliability and robustness should still be improved.

Flexible fluidic actuator is a term used for a wide range of system types, but generically they comprise an expansion chamber defined by an inner wall of an expandable girdle, which is connected to at least two anchoring points. Thus, actuators are able to adapt and transform a fluid pressure force against the inner wall into a traction force or a bending movement. Pneumatic actuators are contractile and linear motion engines are activated by gas pressure. They generally exhibit high power density, but bulky fluidic sources are necessary and miniaturization is limited. A recent review can be found in De Greef et al. (2009).

Cable-driven actuation has the benefit of providing a distributed and continuous action and cables can be fitted at spots within a soft robot where it would be hard to place other actuators otherwise, since powerful motors can be embedded outside the robot thus keeping it flexible. Since cable transmission is continuous and is subject to negligible backlash issues, control is greatly simplified, but friction losses along the robot due to the cables may reduce the controllability of the system. Compared with the other actuation methods, cable actuation offers low inertia, weight, and volume, guarantees fast response times, and long range transmission of force and power.

Other than active actuators, some smart materials have been exploited as semi-active actuators, meaning that they can only dissipate energy during a mechanical interaction. This special class of materials offers the possibility to change its mechanical properties due to controlled physical stimuli. Thermo-, magneto-, and electro-rheological materials possess the capability to change the stiffness from values resembling low viscosity fluids to values similar to solid materials by applying thermal (Cheng et al., 2010), magnetic, or electric fields (Yalcintas and Dai, 1999), respectively. The main drawbacks are due to control issues and low response time (for thermal activation) or the high fields required (for the magnetic and electric activation).

Granular jamming is another phenomenon which is raising a growing interest for the impressive behavior, which enables particles to act like a liquid, solid, or something in between depending on an applied vacuum level (Steltz et al., 2010).

Though the main focus of research in soft robotics is still on materials and actuators, manufacturing such a kind of artifacts represents another very important challenge to face. New manufacturing processes have been developed including Shape Deposition Manufacturing (SDM) and Smart Composite Microstructures (SCM) (Cho et al., 2009). Yet, despite the growing interest and need for this field, currently there is only a handful of example soft robots, which conform to the definition of soft robotics adopted here and most of them integrate only two or three of the components just reported.

## Control Architectures and Paradigms for Soft Robots

As mentioned, the well-known robot control theories and techniques result poorly applicable when robots are built with soft materials and are generally continuum robots (Robinson and Davies, 1999). Most of the approaches currently in use for the direct model of continuum soft robots are limited to piecewise-constant-curvature approximation (Camarillo et al., 2009). Jones et al. (2009) presented a steady state model of continuous robot neglecting the actuation. In the work of Boyer et al. (2006), the distributed force and torque acting on the robot are estimated but no discussion is made concerning on the actuators that could generate them. A continuum geometrically exact approach for tendon-driven continuum robot has been proposed by Renda et al. (2012). It is capable of properly simulating the coupled tendon drive behavior of non-constant curvature manipulators, because it takes into account the torsion of the robot. In Wittmeier et al. (2013), six different control approaches inspired by classical control theory, machine learning, and neuroscience were evaluated in controlling a cable-driven robot. The inverse model proposed in literature for controlling continuum soft robot follows different approaches. A modal approach was proposed by Chirikjian and Burdick (1994). In Giorelli et al. (2012), a successful Jacobian method for a non-constant curvature tendon-driven manipulator is proposed.

On the other hand, the concepts of embodied intelligence and morphological computation can potentially help to control soft robots. The way embodied intelligence can be exploited is by taking into account the interaction with the environment. Different from current approaches, the complex interaction of a soft robot with the environment is not modeled analytically, but it is encoded in internal models, built by learning from experience in the real physical world, similarly to how internal models are built in brains (Laschi et al., 2008). The internal models encode the correlations between sensory and motor data and encode the part of control that is done by the morphology of the body interacting with the environment, i.e., the part of control that is given by morphological computation. The tools are those of soft computing, with special regard to self-organizing neural networks (Asuni et al., 2006).

Recently, neural networks have been employed in continuum robots to learn manipulator configurations from actuator inputs. Interestingly, Giorelli et al. (2013) presented a comparison of the performance of a soft arm controller developed with a neural approach and with an inverse Jacobian approach, showing how the neural control system can take into account the variability of the arm with no effect on the performance.

## First Steps of Soft Robotics

**Figure 1:**
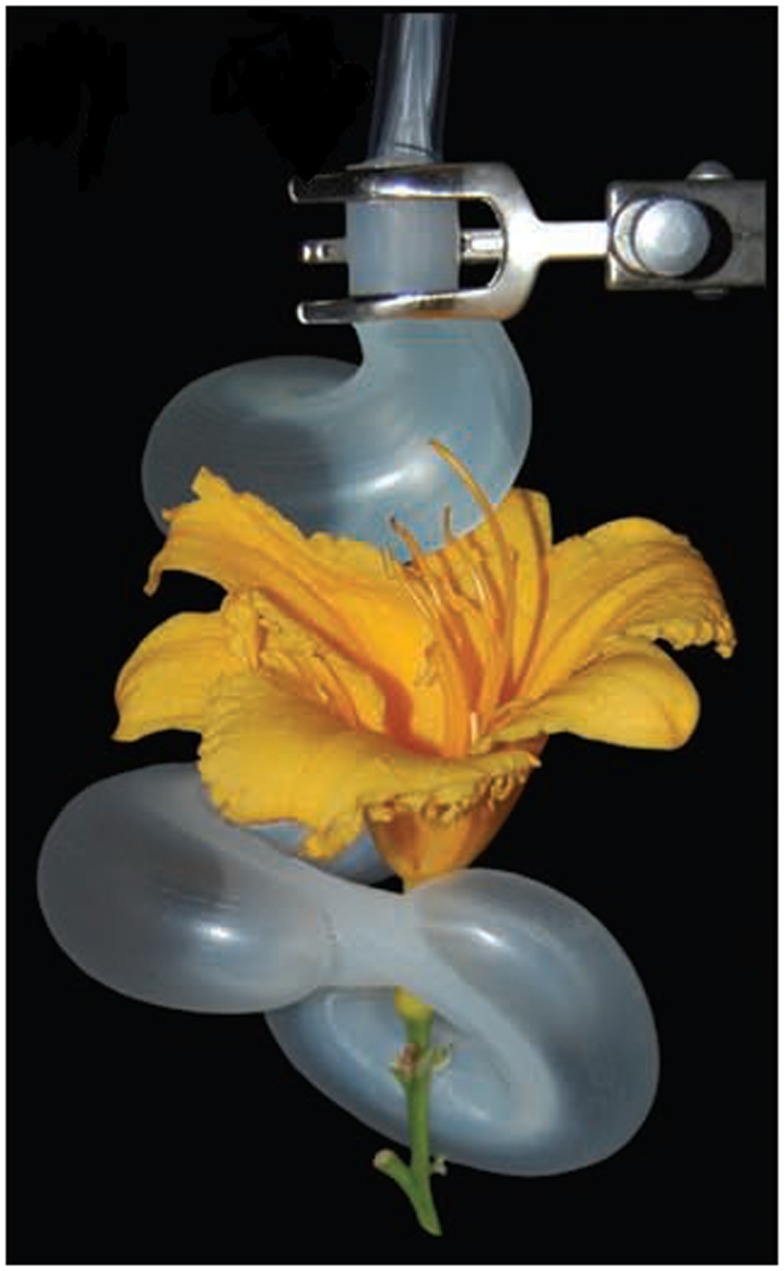
**Pneumatic tentacle-like soft manipulator (Martinez et al., 2013) (reproduced with permission from John Wiley and Sons)**.

**Figure 2:**
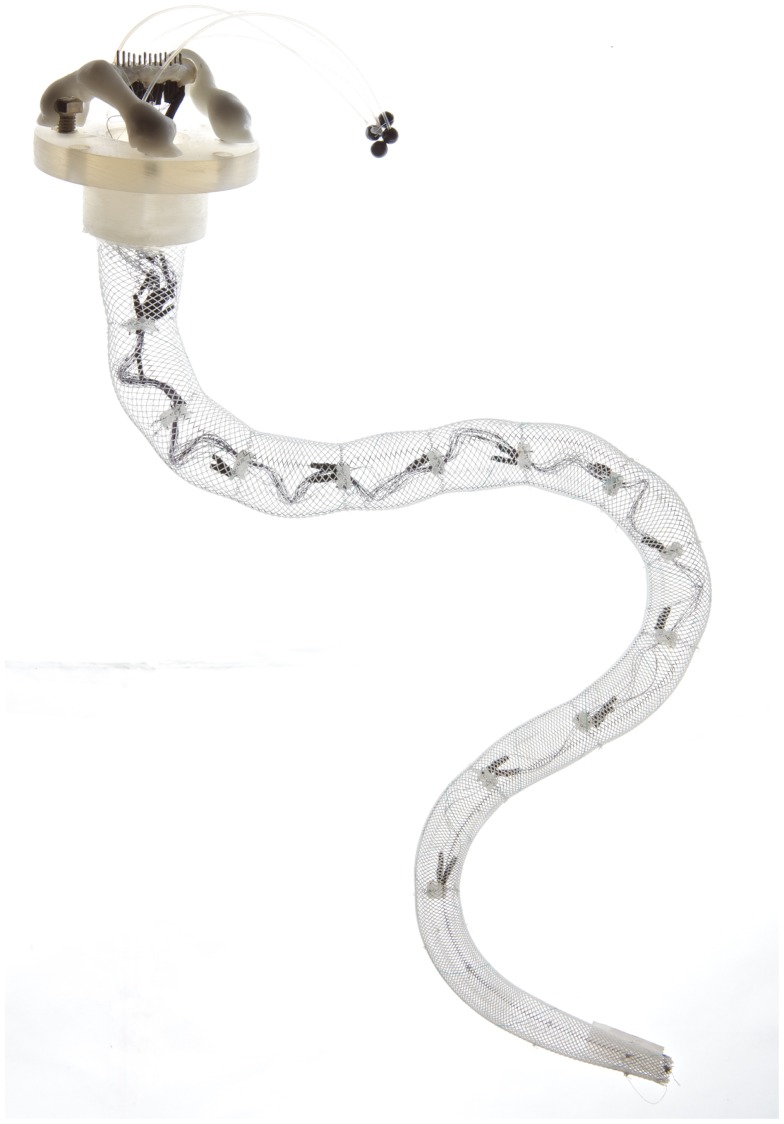
**Flexible octopus-like robot arm, composed by a braided sheath actuated by SMA springs (photo by Massimo Brega, The Lighthouse)**.

At Harvard University, a series of soft robots based on pneumatic actuation has been developed. They entail starfish-like (Shepherd et al., 2011) and tentacle-like robots (Martinez et al., 2013) shown in Figure 1, capable of large deformation and with camouflage ability. In this case, articulated motion of the limbs is generated by a single source of pressure and the movement is based on the selection of the distribution, configuration, and size of an embedded pneumatic network. Among the other silicone-based soft robots, it is worth mentioning the soft caterpillar robot inspired by the *Manduca sexta*, the GoQBot, where SMA actuators and the incompressibility of fluids is exploited to deliver performance resembling those of the hydrostatic skeletons (Trimmer et al., 2006; Lin et al., 2011) and the octopus-inspired robots developed at Scuola Superiore Sant’Anna, where the combination of soft materials and cable-driven transmission enabled manipulation capabilities (Cianchetti et al., 2011), legged locomotion (Calisti et al., 2011), and swimming (Giorgio Serchi et al., 2013). The JamBots (Steltz et al., 2010) are another example of how soft materials in combination with soft actuation technologies can be used for robot locomotion and grasping: while the material properties can be changed with granular jamming (determining anisotropies), motion can be generated with pneumatic actuators or with cable-driven systems as in the case of the MIT jammable manipulator (Cheng et al., 2012). Soft materials can be also be part of the actuation system itself as in the case of the use of EAP in the starfish-like robot (Otake et al., 2002) or in the tissue-engineered multi-limbed medusoid robot (Nawroth et al., 2012). Robots based on the exploitation of flexible structures can be considered soft because they exploit antagonistic arrangement and elastic properties of the flexible materials they are made of. Paradigmatic examples are the Meshworm robots where a series of SMA springs arranged in antagonistic manner supported by a flexible braided mesh-tube structure is used to produce a peristaltic motion (Seok et al., 2012), and the octopus-like arm where dexterous manipulation capabilities are reproduced by artificial muscular hydrostats based on a conical braided sheath that is used as the body of the arm and as a support for the SMA actuation system (Laschi et al., 2012), shown in Figure 2.

Though soft robotics is still in its infancy, and current examples of soft robots may appear as very specific solutions, somehow limited in scope, this field is producing a variety of technological solution that can constitute the building blocks of advanced robots. Soft robotics is not just a new direction of technological development, but a novel approach to robotics, unhinging its fundamentals, with the potential to produce a new generation of robots, in the support of humans in our natural environments.

## Conflict of Interest Statement

The authors declare that the research was conducted in the absence of any commercial or financial relationships that could be construed as a potential conflict of interest.
